# Comparative Analysis of the Mitochondrial Genomes of Three Species of Elmidae (Coleoptera: Dryopoidea)

**DOI:** 10.3390/insects16030247

**Published:** 2025-02-28

**Authors:** Zeliang Qin, Na Li, Yaqi Mo, Juping Wang, Yunfei Peng, Fan Song

**Affiliations:** 1 College of Plant Protection, Shanxi Agricultural University, Taiyuan 030031, China; zeliangqin1997@163.com (Z.Q.); tinnso17@163.com (N.L.); moyaqi2000@163.com (Y.M.); pengyunfei1989@sina.com (Y.P.); 2 Shanxi Key Laboratory of Integrated Pest Management in Agriculture, Taiyuan 030031, China; 3 MOA Key Lab of Pest Monitoring and Green Management, Department of Entomology, College of Plant Protection, China Agricultural University, Beijing 100193, China; fansong@cau.edu.cn

**Keywords:** Elmidae, mitochondrial genome, gene rearrangement, phylogenetic analysis

## Abstract

The mitochondrial genomes of three species of Elmidae were sequenced. The sizes were 16,309 bp (*C. jaechi*), 16,291 bp (*G. longiusculus*), and 15,480 bp (*S. punctulata*). Each genome includes 13 protein-coding genes (PCGs), 22 transfer RNA genes (tRNAs), two ribosomal RNA genes (rRNAs), and a control region (CR). All three mitogenomes show AT bias. Except for *trnS1*, lacking the dihydrouridine (DHU) arm, all tRNA genes had a typical cloverleaf structure. The codon usage preferences of the three species showed high similarity. The arrangement of the genes in the three mitogenomes was consistent among them but differed from that in the typical mitogenomes of Coleoptera. The Ka/Ks ratio indicated that *COX1* and *nad4L* had a lower evolutionary rate, being relatively conserved genes among 13 PCGs. The phylogenetic analysis revealed that Elmidae belongs to the superfamily Dryopoidea within the suborder Polyphaga and is monophyletic. The results preliminarily indicate that the taxonomic groups at the subfamily, tribe, and genus levels within Elmidae are ambiguous, and further revisions may be required in the future.

## Simple Summary

Elmidae (Coleoptera: Dryopoidea) is a widespread family of aquatic beetles that currently contains about 151 genera with approximately 1500 species. The phylogenetic positions of the intrafamilial taxonomic groups remain ambiguous. To further understand the position of Elmidae within Coleoptera and the relationships among the subfamily, tribe, and genus levels, three complete mitochondrial genomes of Elmidae (*Cuspidevia jaechi*, *Grouvellinus longiusculus*, and *Stenelmis punctulata*) were sequenced, annotated, analyzed, and compared. The nucleotide composition, genome organization, and codon usage of the three species were highly similar. However, their gene arrangement differed from that of typical mitochondrial genomes in Coleoptera. Phylogenetic analysis indicates that the higher taxonomic groups within Elmidae require further supplementation. This study contributes to the mitogenomic library of Elmidae, providing a scientific basis for an understanding of the evolution of Elmidae species.

## 1. Introduction

Elmidae (riffle beetles) is a diverse group of aquatic Coleoptera, with about 1500 described species [1,2]. Most elmids inhabit running water, with both larvae and adults attaching to submerged wood or stones [3,4]. The elmid is used as an indicator species in water quality monitoring, as many species are particularly sensitive to their aquatic environments [3,4,5,6]. Elmidae currently contains two subfamilies, 151 genera, and five tribes [2,7,8]. Previous studies of Elmidae have mainly focused on the description and identification of morphological characteristics, while the molecular phylogenetic analysis of this family is still limited. Based on *COX1* and two nuclear genes, the phylogenetic relationships among 58 species from 17 genera of Elmidae were analyzed [9]. Based on 585 ultraconserved elements (UCEs), a phylogenetic analysis of 73 elmid species was performed [2]. The available data on the complete mitochondrial genome of Elmidae are very limited. Currently, the complete mitochondrial genome of this family consists only of *Hydora* sp. (OR414025) and *Stenelmis orthotibiata* (PQ754205), both available in the NCBI database. There is still some controversy over the phylogenetic relationships of higher taxonomic groups within this family [2,9].

The typical insect mitochondrial genome is a double-stranded circular DNA molecule, ranging from 14 to 20 kb in size and containing 37 genes: 13 protein-coding genes (PCGs), 2 rRNAs, and 22 tRNAs [10,11]. In addition, there is a non-coding control region (CR), which is the start site of replication and transcription [12,13]. Owing to their features of fast evolution, low sequence recombination, and conserved genes, mitochondrial genomes have been widely used in the study of species delimitation, phylogenetic analysis, and molecular evolution [14,15,16].

*Cuspidevia jaechi* Bian & Ji 2010, *Grouvellinus longiusculus* Bian & Jäch 2019, and *Stenelmis punctulata* Bollow 1940 are classified within Elminae of Elmidae [17,18]. In this study, the first complete mitogenomes of *C. jaechi*, *G. longiusculus*, and *S. punctulata* were determined and annotated. The genome organization, nucleotide composition, codon usage, tRNA gene secondary structure, and control region were investigated and analyzed. The features of the mitochondrial genomes of the three species were compared. Phylogenetic trees were reconstructed for 56 species (including 54 Coleoptera and 2 outgroups) to verify the phylogenetic position of the family Elmidae within Coleoptera, using the Bayesian inference (BI) and maximum likelihood (ML) methods. This study enriches the mitochondrial database on Elmidae, aiding in the understanding of its mitochondrial genome structure and phylogeny.

## 2. Materials and Methods

### 2.1. Sample Collection, Identification, and DNA Extraction

Adult specimens of *C. jaechi*, *G. longiusculus*, and *S. punctulata* were collected using the water net method in China (Table 1). All specimens were preserved in anhydrous ethanol during collection and then stored at −20 °C at the College of Plant Protection, Shanxi Agricultural University. Total genomic DNA was extracted from the whole insect using an Insect DNA Kit (OMEGA, Norcross, GA, USA), following the manufacturer’s protocol. The DNA was stored at −20 °C for further analysis.

### 2.2. Mitogenome Sequencing, Assembly, and Annotation

The Illumina TruSeq libraries were constructed with an average insert size of 350 bp and sequenced using the Illumina NovaSeq 6000 platform (Berry Genomics, Beijing, China) with 150 bp paired-end reads. Quality control was carried out using Fastp version 0.23.2 [19] on the raw data, and low-quality or short reads were removed. The generated sequence was preliminarily annotated using the MITOS Web Server [20] (http://mitos.bioinf.uni-leipzig.de/index.py, accessed on 10 July 2024) and MitoZ version 3.6 [21] with the invertebrate mitochondrial genetic code for automated annotation. The sequences obtained were annotated in Geneious version 10.3 [22], with the default parameters and the mitogenomes of *Hydora* sp. (OR414025) and *Stenelmis orthotibiata* (PQ754205) as the references. Protein-coding genes (PCGs) and ribosomal RNA (rRNA) genes were annotated by aligning the homologous genes of the reference mitogenomes. Transfer RNA (tRNA) genes were confirmed by the online tRNAscan-SE search server [23] (https://lowelab.ucsc.edu/tRNAscan-SE/index.html, accessed on 15 July 2024). The circular mitogenomic maps were visualized using the CGView server [24] (http://stothard.afns.ualberta.ca/cgview_server/, accessed on 16 July 2024).

### 2.3. Sequence Analyses

According to the annotation of the mitochondrial genome, the gene size and structure were analyzed. The nucleotide composition and relative synonymous codon usage (RSCU) of 13 PCGs were calculated by MEGA version 11.0 [25]. AT-skew and GC-skew were calculated using the formulae AT-skew = (A − T)/(A + T) and GC-skew = (G − C)/(G + C) [26]. The non-synonymous substitution rate (Ka), synonymous substitution rate (Ks), and nucleotide diversity (Pi) of protein-coding genes were calculated by DnaSP v6.12.03 [27]. Secondary structures for tRNAs were manually drawn according to the tRNAscan-SE search server [23] (https://lowelab.ucsc.edu/tRNAscan-SE/index.html, accessed on 15 July 2024) and ARWEN version 1.2 [28]. The tandem repeats in the control region were predicted using the Tandem Repeats Finder [29] (https://tandem.bu.edu/trf/trf.html, accessed on 20 July 2024).

### 2.4. Phylogenetic Analysis

For the phylogenetic analysis, we employed 19 families and 54 species of Coleoptera, including the three newly determined sequences as ingroups. Meanwhile, *Archaeoattacus malayanus* and *Tischeria decidua* from Lepidoptera were selected as outgroups (Table S1). Based on the 13 PCGs and 2 rRNA genes, the phylogenetic analysis of Coleoptera was reconstructed.

The 13 PCGs and 2 rRNA genes of 56 species were aligned using the “G-INS-i” strategy in MAFFT version 7 [30] and trimmed using Gblocks [31] in Phylosuite [32,33]. Then, we concatenated the sequences of the 13 PCGs and 2 rRNA genes to obtain 2 data matrices, (1) PCGs12 + rRNAs and (2) PCGs123 + rRNAs, for phylogenetic analysis. The heterogeneity and substitution saturation were evaluated for each data matrix using AliGROOVE v1.08 and DNAMBE v5 [34,35]. The datasets were partitioned using PartitionFinder 2.1.1 [36] with the “greedy” search algorithm and Bayesian Information Criterion (BIC). Details of the best-fit schemes calculated for each partition are shown in Table S2.

A maximum likelihood (ML) tree was constructed using IQ-TREE v1.6.12 [37] under an edge-linked partition model. Bootstrap support (BS) was assessed using 5000 ultrafast bootstrap (UFB) replicates [38]. We used PhyloBayes MPI v.1.5a [39] for BI analyses under the site-heterogeneous mixture model CAT + GTR with a discrete gamma distribution and four rate categories. Two Markov Monte Carlo chains were run independently until the sampled trees reached satisfactory convergence (maxdiff less than 0.1). After the initial 25% of trees were discarded as burn-in, a consensus tree was computed from the remaining trees combined from two runs. iTOL v6 [40] was used to visualize the phylogenetic trees of ML and BI.

## 3. Results

### 3.1. Mitogenome Features of the Three Elmidae Species

**Figure 1:**
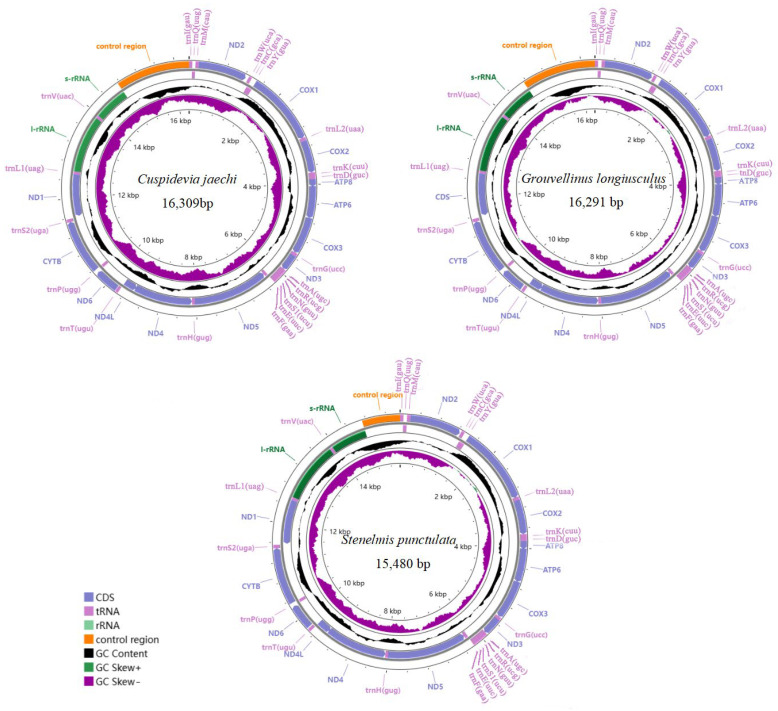
Mitochondrial genome maps of *C. jaechi*, *G. longiusculus*, and *S. punctulata*. Plots of GC content and skew use a window size of 500 and reflect the GC content/skew scores on a scale of 0 to 1 using a baseline of 0.5. Positive and negative skew is indicated by values above and below the midpoint, respectively.

The complete mitogenomes of *C. jaechi*, *G. longiusculus*, and *S. punctulata* were 16,309 bp, 16,291 bp, and 15,480 bp in length, respectively (Figure 1). The differences in the lengths of the mitochondrial genomes were mainly due to differences in the lengths of the control regions. The mitochondrial genomes of three Elmidae species contained 37 genes (13 PCGs, 2 rRNAs, and 22 tRNAs) and a non-coding control region (CR). Among these 37 genes, the J-strand encoded 23 genes, including 14 tRNA genes and 9 PCGs. There were 14 genes on the N-strand, including 4 PCGs, 8 tRNA genes, and 2 rRNA genes (Tables S3–S5).

The three species harbor gene overlap regions, with 13 gene overlaps in *C. jaechi*, 14 in *G. longiusculus,* and 20 in *S. punctulata*. The longest gene overlap regions of *C. jaechi* and *G. longiusculus* are both located in *trnW*/*trnC* and *trnY*/*COX1*, which is 8 bp. The longest overlap region of *S. punctulata* is located in *trnY*/*COX1*, with 9 bp. There are intergenic spacers in the three species, with seven intergenic spacers in *C. jaechi*, nine in *G. longiusculus,* and six in *S. punctulata*. The longest intergenic spacer of *C. jaechi* is located in *nad2*/*trnW*, which is 52 bp. The longest intergenic spacers of *G. longiusculus* and *S. punctulata* are both located in *trnT*/*nad6*, with 24 and 20 bp, respectively (Tables S3–S5).

The nucleotide composition of *C. jaechi* is A = 42.0%, T = 33.0%, G = 8.8%, and C = 16.3%; that of *G. longiusculus* is A = 41.9%, T = 34.2%, G = 9.6%, and C = 14.3%; and that of *S. punctulata* is A = 42.0%, T = 34.1%, G = 9.2%, and C = 14.7% (Table 2). The nucleotide compositions of the three species possess significant AT bias, ranging from 74.9% (*C. jaechi*) to 76.1% (*G. longiusculus* and *S. punctulata*). The A + T content of the control region is the highest; the PCGs have the lowest AT content. The AT content in rRNAs is higher than that in tRNAs and PCGs in the three species (Table 2). The AT bias is positive and the GC bias is negative in the mitogenomes of the three species. The AT and GC biases in protein-coding genes were both negative, while the genes on the J-strand were both negative, and the N-strand had negative AT and positive GC (Table 2).

### 3.2. Protein-Coding Genes (PCGs) and Codon Usage

The PCGs of *C. jaechi*, *G. longiusculus*, and *S. punctulata* are 11,184 bp, 11,178 bp, and 11,188 bp in size, respectively (Table 2). The *nad1* of the three species begins with TTG as the start codon, and the other 12 PCGs start with ATN codons (ATA, ATC, ATG, ATT). Among the 13 PCGs, most of the PCGs used complete stop codons, namely TAA or TAG. A few genes used a single T as an incomplete stop codon. *COX2*, *COX3,* and *nad5* in *C. jaechi* and *G. longiusculus* and *COX2*, *COX3*, *nad5,* and *nad4* in *S. punctulata* used a single T as a stop codon (Tables S3–S5). These incomplete termination codons are presumed to be filled by polyadenylation during the mRNA maturation process [41].

**Figure 2:**
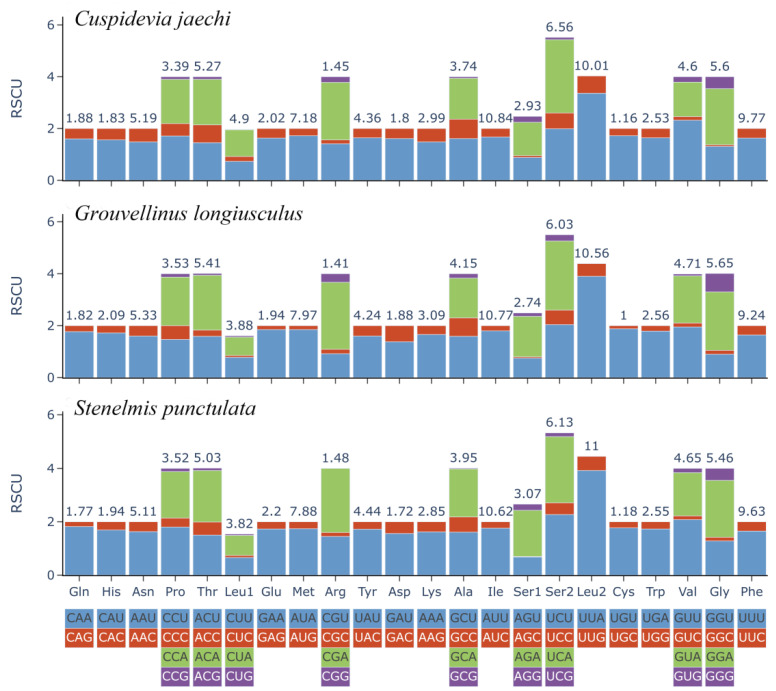
Relative synonymous codon usage (RSCU) of *C. jaechi*, *G. longiusculus*, and *S. punctulata* mitogenomes. The number above the bar graph indicates the frequency of amino acids. The number of codons per amino acid varies from 2 to 4. The RSCU values are color-coded based on the codons below the amino acid labels.

The relative synonymous codon usage (RSCU) of the three species also indicates a strong AT bias in the mitogenome nucleotide composition (Figure 2). TTA, ATT, TTT, ATA, and AAT are the most frequently used codons in these three species. The usage frequency of TTA was the highest in *G. longiusculus* and *S. punctulata*, while ATT was used most frequently in *C. jaechi*. The most common amino acid was Ile, followed by Leu2, Phe, and Met, in *C. jaechi* and *G. longiusculus*, while the most used amino acid was Leu2, followed by Ile, Phe, and Met, in *S. punctulata* (Figure 2).

### 3.3. Transfer RNAs and Ribosomal RNA Genes

**Figure 3:**
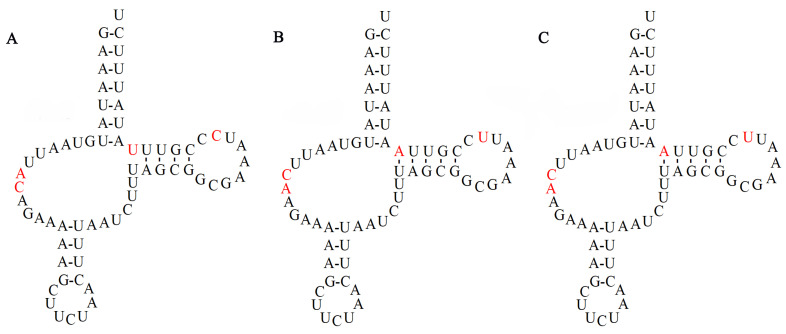
Predicted secondary structure of *trnS1* in *C. jaechi*, *G. longiusculus*, and *S. punctulata*. Bases that differ among the three are shown in red. (**A**) *C. jaechi*, (**B**) *G. longiusculus*, (**C**) *S. punctulata*.

The mitogenomes of the three species contain 22 tRNA genes, which can transport all 20 amino acids. The 14 tRNAs were located on the J-strand, and 8 tRNAs were encoded on the N-strand. The lengths of tRNA genes ranged from 63 bp (*trnC*, *trnL1*) to 71 bp (*trnK*, *trnV*) in *C. jaechi*, from 61 bp (*trnC*) to 71 bp (*trnK*) in *G. longiusculus*, and from 61 bp (*trnC*) to 76 bp (*trnQ*) in *S. punctulata* (Tables S3–S5). The secondary structure of *trnS1* lacked the dihydrouridine (DHU) arm (Figure 3), which is common in metazoan mitogenomes [14]; the other tRNAs had a typical cloverleaf secondary structure (Figures S1–S3). In addition, the anticodon of *trnS1* is UCU (Figure 3), which differs from the common GCU; such a situation was supposed to be a synapomorphy of the Polyphaga [42]. GU, AG, UU, and UC mismatches were found in the three species, and the number of GU mismatches was the highest in all three species. AG mismatches were found in the *trnW* of the three species, UU mismatches were found in the *trnI* of *C. jaechi* and *trnL2* of *G. longiusculus*, and UC mismatches were only found in the *trnN* of *C. jaechi* (Figures S1–S3).

The rRNA genes of three mitogenomes were encoded on the N-strand. *l-rRNA* is located between *trnL1* and *trnV*, and *s-rRNA* is located between *trnV* and the control region (Figure 1) (Tables S3–S5). The length of *l-rRNA* is 1298 bp in *C. jaechi*, 1306 bp in *G. longiusculus,* and 1296 bp in *S. punctulata*. The length of *s-rRNA* is 778 bp in *C. jaechi*, 776 bp in *G. longiusculus,* and 777 bp in *S. punctulata*. The two rRNAs contain a high AT bias (Table 2).

### 3.4. Control Region

The control region (CR) is the main non-coding region of the mitogenome and is located between the *s-rRNA* gene and *trnI*. This region is associated with the origin of replication and transcription [11,14]. The lengths of the CR were 1553 bp in *C. jaechi*, 1543 bp in *G. longiusculus*, and 753 bp in *S. punctulata*. The AT content of this region was the highest, at 80.9% (*C. jaechi*), 82.8% (*G. longiusculus*), and 87.0% (*S. punctulata*) (Table 2).

Tandem duplication in the CR has been reported in other sequenced insect mitogenomes [43]. The lengths and numbers of the tandem repeats in the three species were different. There were two tandem repeats in the CRs of *C. jaechi* and *G. longiusculus*. Three were found in *S. punctulata*. Moreover, microsatellite-like (AT)_n_ and (CT)_n_ elements were found in the CR. (AT)_7_, (AT)_5_, (AT)_9_, and (CT)_6_ were found in *C. jaechi*; (AT)_6_, (AT)_8_, and (CT)_5_ were found in *G. longiusculus*; and (TA)_5_ was found in *S. punctulata*. Additionally, there were some poly-A and poly-T structures in the CRs of the three species. There were three poly-As (6 bp, 9 bp, and 7 bp) and three poly-Ts (7 bp, 10 bp, and 10 bp) in *C. jaechi*. Four poly-As (7 bp, 8 bp, 7 bp, and 10 bp) and four poly-Ts (14 bp, 7 bp, 10 bp, and 11 bp) were found in *G. longiusculus*. Two poly-As (7 bp and 9 bp) and one poly-T (10 bp) were found in *S. punctulata* (Figure S4).

### 3.5. Nucleotide Diversity and Evolutionary Rate Analysis

**Figure 4:**
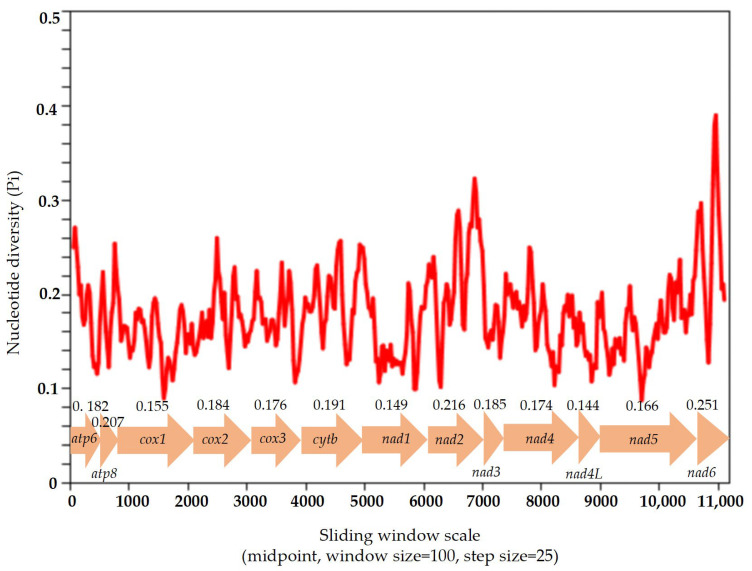
Nucleotide diversity of 13 PCGs in Elmidae. Sliding window analysis of the alignment of 13 protein-coding genes. The value of the nucleotide diversity (Pi) is indicated by the red curve. Pi values and genes are indicated below the red curve.

Based on the 13 aligned PCGs of five sequenced Elmidae species, the sliding window analysis showed that the nucleotide diversity values ranged from 0.144 (*nad4L*) to 0.251 (*nad6*) (Figure 4). The *nad6* gene is the most variable region with the highest nucleotide diversity (Pi = 0.251), followed by *nad2* (Pi = 0.216) and *atp8* (Pi = 0.207). The *nad4L* gene exhibited the lowest diversity values (Pi = 0.143), followed by *COX1* (Pi = 0.155), which indicated that these were relatively conserved genes among the 13 PCGs (Figure 4).

**Figure 5:**
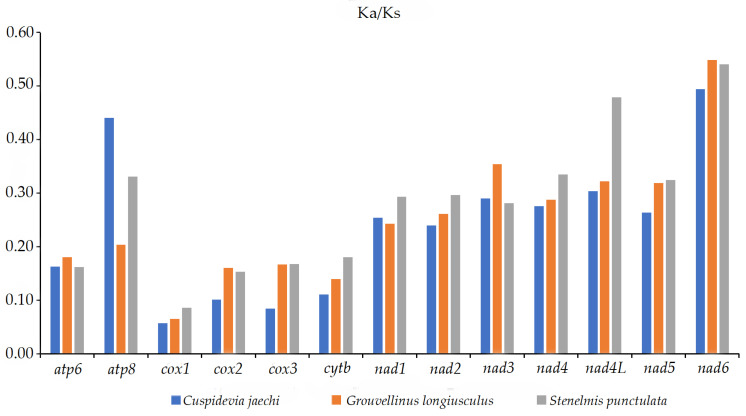
Ka/Ks values of 13 PCGs in three species of Elmidae. The bar indicates each gene’s Ka/Ks value.

The evolutionary rates of the 13 PCGs were estimated using the ratio of Ka/Ks. A ratio of Ka/Ks of less than 1 shows that the genes are under negative (purifying) selection, a value equal to 1 shows neutral evolution, and a value greater than 1 shows positive (adaptative) selection. The Ka/Ks ratios for the 13 PCGs were less than 1, indicating that these genes evolved under purifying selection. The Ka/Ks ratio of *nad6* was the highest, which indicated that the evolutionary rate of *nad6* was the fastest among the 13 PCGs, followed by *nad4L* and *atp8*. *COX1* showed the lowest evolutionary rate, indicating that it was the slowest to evolve and the most conserved gene among the 13 PCGs, followed by *COX3* and *COX2* (Figure 5).

### 3.6. Heterogeneity and Substitution Saturation Tests

**Figure 6:**
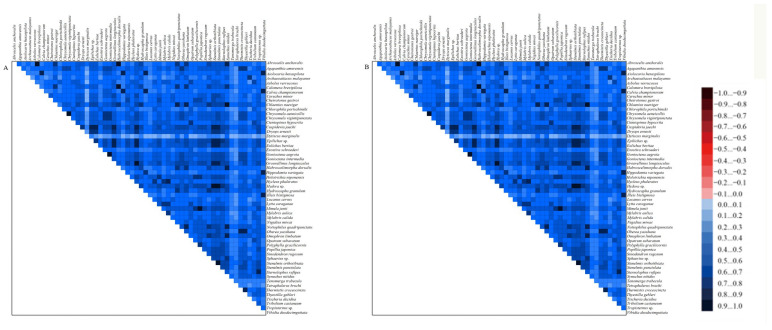
Heterogeneity of the sequence compositions of the mitochondrial genomes in different datasets. The pairwise Aliscore values are indicated by colored squares. Darker colors indicate full random similarity, and lighter colors indicate the opposite. (**A**) PCGs12 + rRNA matrix. (**B**) PCGs123 + rRNA matrix.

Pairwise comparisons of the nucleotide divergence within multiple sequence alignment indicated low heterogeneity across the two matrices. The results indicated that the genetic variation was sufficiently uniform to support accurate evolutionary inferences (Figure 6). The analysis of the two datasets revealed that the simple index of substitution saturation (ISS) was less than the critical ISS value (ISS.c), and *p* < 0.05 (Tables S6 and S7). This suggested that the datasets had not reached saturation in terms of nucleotide substitutions.

### 3.7. Phylogenetic Relationships

**Figure 7:**
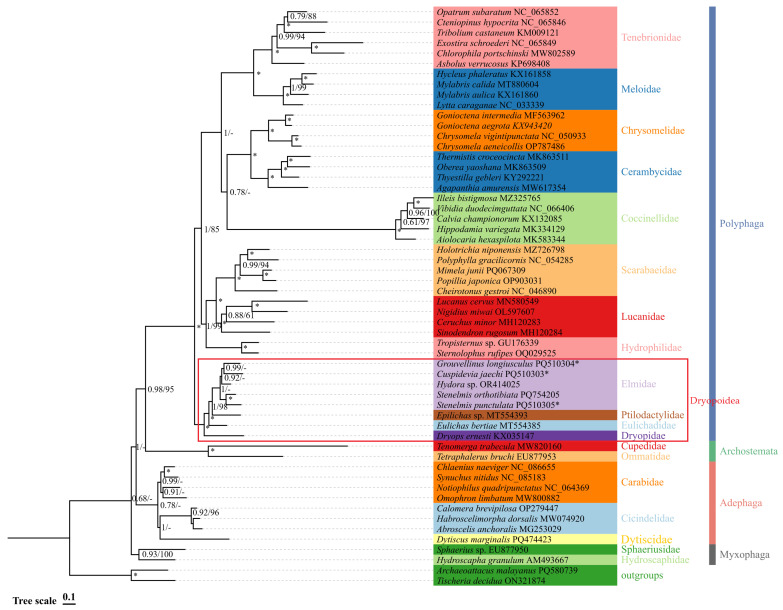
Phylogenetic tree of Elmidae based on the PCGs12 + rRNA matrix obtained using PhyloBayes and IQ-TREE. The numbers on the branches are Bayesian posterior probabilities (PP (**left**)) and bootstrap values (BS (**right**)). “-” indicates that the clades are different. A star symbol indicates that the two methods produced the maximum support value. The species marked with * were the research objects in this study.

**Figure 8:**
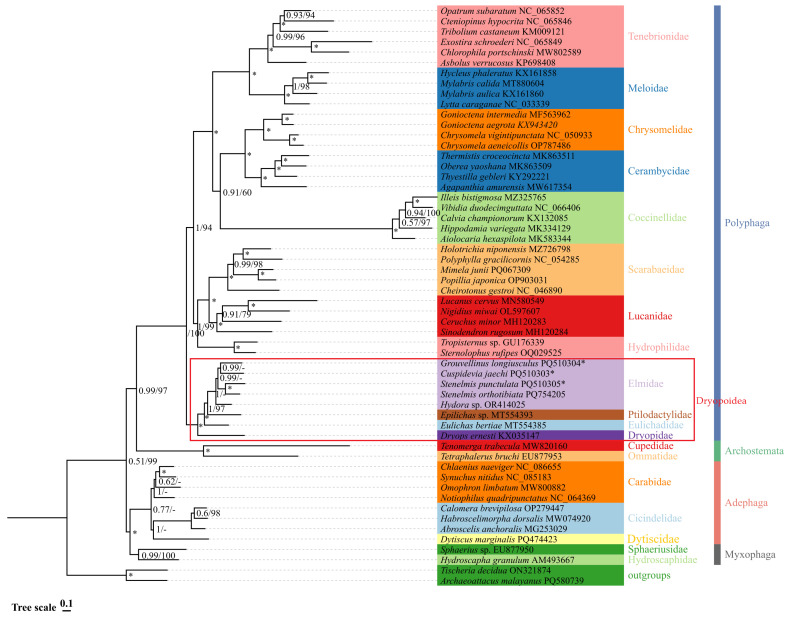
Phylogenetic tree of Elmidae based on the PCGs123 + rRNA matrix obtained using PhyloBayes and IQ-TREE. The numbers on the branches are Bayesian posterior probabilities (PP (**left**)) and bootstrap values (BS, (**right**)). “-” indicates that the clades are different. A star symbol indicates that the two methods produced the maximum support value. The species marked with * were the research objects in this study.

In this study, four phylogenetic trees were constructed via the maximum likelihood (ML) and Bayesian inference (BI) methods using two datasets: PCGs12 + rRNAs and PCGs123 + rRNAs. The four phylogenetic trees exhibited broadly similar topologies (Figure 7 and Figure 8).

The phylogenetic relationships among the suborders within Coleoptera are as follows: except for the Bayesian inference (BI) tree based on PCGs12 + rRNAs, which supports (Myxophaga + (Adephaga + (Archostemata + Polyphaga))), the remaining three trees support (Myxophaga + Adephaga) + (Polyphaga + Archostemata). Each family in this study is consistently supported as a monophyletic group by all four phylogenetic trees, which have high bootstrap support values (Figure 7 and Figure 8).

The four phylogenetic trees consistently support Elmidae belonging to the superfamily Dryopoidea within the suborder Polyphaga, with high support values. The phylogenetic relationship of Polyphaga has two topologies in four phylogenetic trees. Based on the Bayesian inference (BI) tree of PCGs12 + rRNAs, and the BI and maximum likelihood (ML) trees of PCGs123 + rRNAs, the topology was (Dryopidae + (Eulichadidae + (Ptilodactylidae + Elmidae))) + ((Hydrophilidae + (Lucanidae + Scarabaeidae)) + ((Tenebrionidae + Meloidae) + (Coccinellidae + (Cerambycidae + Chrysomelidae)))). The other topology was (Dryopidae + (Eulichadidae + (Ptilodactylidae + Elmidae))) + ((Hydrophilidae + (Lucanidae + Scarabaeidae)) + (Coccinellidae + ((Cerambycidae + Chrysomelidae) + (Tenebrionidae + Meloidae)))), which was based on the ML tree of PCGs12 + rRNAs. Among the two topologies, Coccinellidae was placed in different positions (Figure 7 and Figure 8).

In the four phylogenetic trees, the interspecific relationships of the Elmidae showed variation. The BI tree based on PCGs12 + rRNAs supported (*S. orthotibiata* + *S. punctulata*) + (*Hydora* sp. + (*C. jaechi* + *G. longiusculus*)), and the BI tree based on PCGs123 + rRNAs supported *Hydora* sp. + ((*S. orthotibiata* + *S. punctulata*) + (*C. jaechi* + *G. longiusculus*)). The ML tree based on PCGs12 + rRNAs supported *C. jaechi* + ((*S. orthotibiata* + *S. punctulata*) + (*Hydora* sp. + *G. longiusculus*))*,* while the ML tree based on PCGs123 + rRNAs supported (*G. longiusculus* + *Hydora* sp.) + (*C. jaechi* + (*S. orthotibiata* + *S. punctulata*)). All phylogenetic trees supported the notion that *S. orthotibiata* and *S. punctulata* first gathered into a cluster, which is consistent with the traditional taxonomy [44] (Figure 7 and Figure 8).

## 4. Discussion

The gene directions and arrangements in the three species of Elmidae were found to be consistent with the sequenced species of Dryopoidea (KX035147, MT554385, MT554393), differing notably from most other species of Coleoptera [45]. The primary difference observed was the rearrangement of *trnP* and *nad6*. An alteration in the positions between *nad6* and *trnP* was found in the evolutionary branches of Dryopoidea; this rearrangement can be traced back to the Jurassic period [46].

The phylogenetic analysis indicates that the superfamily Dryopoidea may have diverged earlier from within Polyphaga, which is consistent with the results of the studies by McKenna et al. [47] and Cai et al. [48]. Elmidae was a sister group to Ptilodactylidae. This result differs from the research of Bocak et al. [49], which was based on datasets of *COX1*, *l-rRNA*, *18S*, and *28S* rRNA.

Regarding the phylogenetic relationships within the Elmidae, the results of this study are not consistent with the traditional taxonomy [44]. The taxonomic groups at the subfamily, tribe, and genus levels were ambiguous; this result is consistent with the findings of Hayashi et al. [9] and Kobayashi et al. [2]. At the subfamily level, except for the Bayesian inference (BI) tree based on PCGs123 + rRNAs, all trees showed that the subfamily Larainae (*Hydora* sp.) was nested in the subfamily Elminae group (*S. orthotibiata*, *S. punctulata*, *C. jaechi*, and *G. longiusculus*). At the tribe level, the three species of Elmini (*S. orthotibiata*, *S. punctulata*, and *G. longiusculus*) did not first form a cluster, as supported by the four trees. At the genus level, the phylogenetic relationship varied across the four phylogenetic trees due to the changing positions of *Hydora* sp. and *C. jaechi*.

Currently, molecular research on Elmidae is limited, with only two complete mitochondrial genome sequences available in the NCBI database. This study enhances the information available on Elmidae in the NCBI database and provides a scientific basis for species identification and the evolution of Elmidae insects. The samples and molecular data were limited in this study; future analyses should include more taxa and taxonomic information to facilitate the comprehensive revision of Elmidae.

## 5. Conclusions

In this study, the complete mitogenomes of *C. jaechi*, *G. longiusculus,* and *S. punctulata* were sequenced, analyzed, and compared. It was found that the gene arrangements of these three mitogenomes were different from those of other typical mitogenomes in Coleoptera. The phylogeny of 54 species of Coleoptera was analyzed using the Bayesian inference and maximum likelihood methods. The phylogenetic analysis indicated that Elmidae subordinate to the superfamily Dryopoidea within the suborder Polyphaga, was monophyletic. However, the taxonomic groups at the subfamily, tribe, and genus levels were unstable. This result initially suggests that the classification within Elmidae needs to be confirmed by increasing the number of taxon samples and incorporating more molecular and morphological information in future research.

## Figures and Tables

**Table 1:** The voucher information of the specimens used for mitochondrial genome sequencing in this study.

Specimen	Date of Collection	Collection Site	Longitude (E)	Latitude (N)	GB Number
*C. jaechi*	15 October 2023	Jiulianshan Natural Reserve, Jiangxi	114.504167	24.591389	PQ510303
*G. longiusculus*	9 October 2023	Yunju Mountains, Jiangxi	115.583611	29.093889	PQ510304
*S. punctulata*	25 September 2023	Mount Li, Shanxi	111.993611	35.355000	PQ510305

**Table 2:** Nucleotide compositions of the whole mitogenomes of *C. jaechi*, *G. longiusculus,* and *S. punctulata*.

Species	Type	Length (bp)	A%	T%	G%	C%	A + T%	G + C%	AT-Skew	GC-Skew
*C. jaechi*	PCGs	11,184 (68.6%)	31.6	41.7	12.6	14.2	73.2	26.8	−0.1382	−0.0601
	tRNAs	1452 (8.9%)	38.4	37.9	13.2	10.5	76.2	23.8	0.0063	0.1130
	rRNAs	2076 (12.7%)	32.5	46.0	14.9	6.6	78.5	21.5	−0.1713	0.3870
	l-rRNA	1298 (8.0%)	46.6	32.6	6.2	14.6	79.2	20.8	0.1770	−0.4000
	s-rRNA	778 (4.8%)	44.9	32.4	7.2	15.6	77.2	22.8	0.1614	−0.3672
	control region	1553 (9.5%)	45.0	35.9	6.2	12.9	80.9	19.1	0.1122	−0.3514
	J-strand	7816 (47.9%)	35.7	36.2	10.6	17.5	71.9	28.1	−0.0073	−0.2476
	N-strand	6896 (42.3%)	28.6	48.3	15.7	7.4	76.9	23.1	−0.2570	0.3610
	full genome	16,309	42.0	33.0	8.8	16.3	74.9	25.1	0.1202	−0.3009
*G. longiusculus*	PCGs	11,178 (68.6%)	32.5	42.0	12.5	13.0	74.5	25.5	−0.1273	−0.0196
	tRNAs	1458 (8.9%)	39.4	37.9	13.0	9.7	77.3	22.7	0.0204	0.1420
	rRNAs	2082 (12.8%)	32.9	46.1	14.1	6.9	79.0	21.0	−0.1679	0.3425
	l-rRNA	1306 (8.0%)	46.7	32.9	6.4	13.9	79.6	20.4	0.1731	−0.3684
	s-rRNA	776 (4.8%)	45.1	32.7	7.7	14.4	77.8	22.2	0.1589	−0.3023
	control region	1543 (9.5%)	44.8	38.0	7.3	9.9	82.8	17.2	0.0829	−0.1472
	J-strand	7809 (47.9%)	36.1	37.3	11.4	15.2	73.4	26.6	−0.0154	−0.1419
	N-strand	6909 (42.4%)	30.0	47.7	14.3	8.0	77.7	22.3	−0.2283	0.2824
	full genome	16,291	41.9	34.2	9.6	14.3	76.1	23.9	0.1005	−0.2001
*S. punctulata*	PCGs	11,188 (72.3%)	32.5	42.2	12.3	13.0	74.7	25.3	−0.1306	−0.0258
	tRNAs	1479 (9.6%)	38.5	38.7	13.3	9.5	77.2	22.8	−0.0035	0.1632
	rRNAs	2071 (13.4%)	33.5	45.9	14.1	6.5	79.4	20.6	−0.1557	0.3677
	l-rRNA	1296 (8.4%)	46.4	33.8	6.3	13.6	80.2	19.8	0.1569	−0.3696
	s-rRNA	777 (5.0%)	45.0	33.1	6.9	14.9	78.1	21.9	0.1532	−0.3647
	control region	753 (4.9%)	47.4	39.6	4.9	8.1	87.0	13.0	0.0901	−0.2449
	J-strand	7827 (50.6%)	36.1	37.2	11.1	15.5	73.3	26.7	−0.0159	−0.1648
	N-strand	6911 (44.6%)	30.0	48.2	14.4	7.4	78.2	21.8	−0.2332	0.3201
	full genome	15,480	42.0	34.1	9.2	14.7	76.1	23.9	0.1037	−0.2308

## Data Availability

The genomic data of this study are available under the NCBI accession numbers PQ510303, PQ510304, and PQ510305.

## Supplementary Materials

The following supporting information can be downloaded at: https://www.mdpi.com/article/10.3390/insects16030247/s1, Table S1. NCBI numbers of specimens used for phylogenetic analysis. Table S2. Best partitioning scheme and nucleotide substitution models for different datasets selected by PartitionFinder. Table S3. Mitochondrial genome structure of *Cuspidevia jaechi.* Table S4. Mitochondrial genome structure of *Grouvellinus longiusculus.* Table S5. Mitochondrial genome structure of *Stenelmis punctulata.* Table S6. Substitution Saturation test of PCGs12 + rRNAs matrix. Table S7. Substitution Saturation test of PCGs123 + rRNAs matrix. Figure S1. Predicted secondary structures of tRNAs in *Cuspidevia jaechi.* Figure S2. Predicted secondary structures of tRNAs in *Grouvellinus longiusculus.* Figure S3. Predicted secondary structures of tRNAs in *Stenelmis punctulata.* Figure S4. Control regions of *C. jaechi*, *G. longiusculus*, and *S. punctulata*.
