# Supplementary material for: Comparative Analysis of the Mitochondrial Genomes of Three Species of Elmidae (Coleoptera: Dryopoidea)
Source: Insects. 2025 Feb 28;16(3):247. doi: 10.3390/insects16030247 (PMC11942656; doi:10.3390/insects16030247)
Supplement: Supplementary file 1 [file insects-16-00247-s001.zip › insects-3465974-supplementary.pdf]

Supplementary Mateirals

Comparative Analysis of the Mitochondrial Genomes of Three Species of Elmidae (Coleoptera: Dryopoidea)

Zeliang Qin [1], Na Li [1], Yaqi Mo [1], Juping Wang [1,2,*], Yunfei Peng [1,2] and Fan Song [3]

Table S1. Specimens NCBI number used for phylogenetic analysis.

	Family	Species	GeneBank ID
ingroups	Carabidae	*Chlaenius naeviger*	NC_086655
		Notiophilus quadripunctatus	NC_064369
		Omophron limbatum	MW800882
		Synuchus nitidus	NC_085183
	Cerambycidae	*Agapanthia amurensis*	MW617354
		Oberea yaoshana	MK863509
		Thermistis croceocincta	MK863511
		Thyestilla gebleri	KY292221
	Chrysomelidae	*Chrysomela aeneicollis*	OP787486
		Chrysomela vigintipunctata	NC_050933
		Gonioctena aegrota	KX943420
		Gonioctena intermedia	MF563962
	Cicindelidae	*Abroscelis anchoralis*	MG253029
		Calomera brevipilosa	OP279447
		Habroscelimorpha dorsalis	MW074920
	Coccinellidae	*Aiolocaria hexaspilota*	MK583344
		Calvia championorum	KX132085
		Hippodamia variegata	MK334129
		Illeis bistigmosa	MZ325765
		Vibidia duodecimguttata	NC_066406
	Cupedidae	*Tenomerga trabecula*	MW820160
	Dryopidae	*Dryops ernesti*	KX035147
	Dytiscidae	*Dytiscus marginalis*	PQ474423
	Elmidae	*Cuspidevia jaechi*	PQ510303
		Grouvellinus longiusculus	PQ510304
		Hydora sp.	OR414025
		Stenelmis orthotibiata	PQ754205
		Stenelmis punctulata	PQ510305
	Eulichadidae	*Eulichas bertiae*	MT554385
	Hydrophilidae	*Sternolophus rufipes*	OQ029525
		Tropisternus sp.	GU176339
	Hydroscaphidae	*Hydroscapha granulum*	AM493667
	Lucanidae	*Ceruchus minor*	MH120283
		Lucanus cervus	MN580549
		Nigidius miwai	OL597607
		Sinodendron rugosum	MH120284
	Meloidae	*Hycleus phaleratus*	KX161858
		Lytta caraganae	NC_033339
		Mylabris aulica	KX161860
		Mylabris calida	MT880604
	Ommatidae	*Tetraphalerus bruchi*	EU877953
	Ptilodactylidae	*Epilichas* sp.	MT554393

	Scarabaeidae	*Cheirotonus gestroi*	NC_046890
		Holotrichia niponensis	MZ726798
		Mimela junii	PQ067309
		Polyphylla gracilicornis	NC_054285
		Popillia japonica	OP903031
	Sphaeriusidae	*Sphaerius* sp.	EU877950
	Tenebrionidae	*Asbolus verrucosus*	KP698408
		Chlorophila portschinski	MW802589
		Cteniopinus hypocrita	NC_065846
		Exostira schroederi	NC_065849
		Opatrum subaratum	NC_065852
		Tribolium castaneum	KM009121
Outgroups		*Archaeoattacus malayanus*	PQ580739
		Tischeria decidua	ON321874

Table S2. Best partitioning scheme and nucleotide substitution models for different datasets selected by PartitionFinder.

Dataset	Partition	Subset partitions	Best model
PCGs12+rRNAs-BI	P1	atp6_codonA, cytb_codonA	GTR+I+G+X
	P2	atp6_codonB, cox2_codonB	GTR+I+G+X
	P3	atp8_codonA, nad6_codonA	HKY+I+G+X
	P4	atp8_codonB	GTR+I+G+X
	P5	cox1_codonA	GTR+I+G+X
	P6	cox1_codonB	GTR+I+G+X
	P7	cox2_codonA	GTR+I+G+X
	P8	cox3_codonA	GTR+I+G+X
	P9	cox3_codonB	GTR+I+G+X
	P10	cytb_codonB	GTR+I+G+X
	P11	nad1_codonA	GTR+I+G+X
	P12	nad1_codonB	GTR+I+G+X
	P13	nad2_codonA	GTR+I+G+X
	P14	nad3_codonB, nad2_codonB	GTR+I+G+X
	P15	nad3_codonA	GTR+I+G+X
	P16	nad4L_codonA	GTR+I+G+X
	P17	nad4L_codonB	GTR+I+G+X
	P18	nad4_codonA, nad5_codonA	GTR+I+G+X
	P19	nad4_codonB	GTR+I+G+X
	P20	nad5_codonB	GTR+I+G+X
	P21	nad6_codonB	GTR+I+G+X
	P22	rrnL	TRN+I+G+X
	P23	rrnS	GTR+I+G+X
PCGs12+rRNAs-ML	P1	atp6_codonA, cytb_codonA	GTR+F+I+G4
	P2	atp6_codonB, cox2_codonB	GTR+F+I+G4
	P3	atp8_codonA, nad6_codonA	GTR+F+I+G4
	P4	atp8_codonB	GTR+F+I+G4
	P5	cox1_codonA	GTR+F+I+G4
	P6	cox1_codonB	GTR+F+I+G4
	P7	cox2_codonA	GTR+F+I+G4
	P8	cox3_codonA	GTR+F+I+G4
	P9	cox3_codonB	GTR+F+I+G4
	P10	cytb_codonB	GTR+F+I+G4
	P11	nad1_codonA	GTR+F+I+G4
	P12	nad1_codonB	GTR+F+I+G4

	P13	nad2_codonA	GTR+F+I+G4
	P14	nad3_codonB, nad2_codonB	GTR+F+I+G4
	P15	nad3_codonA	GTR+F+I+G4
	P16	nad4L_codonA	GTR+F+I+G4
	P17	nad4L_codonB	GTR+F+I+G4
	P18	nad4_codonA, nad5_codonA	GTR+F+I+G4
	P19	nad4_codonB	GTR+F+I+G4
	P20	nad5_codonB	GTR+F+I+G4
	P21	nad6_codonB	GTR+F+I+G4
	P22	rrnL	GTR+F+I+G4
	P23	rrnS	GTR+F+I+G4
PCGs123+rRNAs-BI	P1	atp6_codon1, cytb_codon1	GTR+I+G+X
	P2	cox2_codon2, atp6_codon2	GTR+I+G+X
	P3	atp6_codon3, atp8_codon3	TRN+I+G+X
	P4	nad6_codon1, atp8_codon1	GTR+I+G+X
	P5	atp8_codon2	GTR+I+G+X
	P6	cox1_codon1	GTR+I+G+X
	P7	cox1_codon2	GTR+I+G+X
	P8	cox1_codon3	GTR+I+G+X
	P9	cox2_codon1	GTR+I+G+X
	P10	cox2_codon3, cox3_codon3	GTR+I+G+X
	P11	cox3_codon1	GTR+I+G+X
	P12	cox3_codon2	GTR+I+G+X
	P13	cytb_codon2	GTR+I+G+X
	P14	nad3_codon3, cytb_codon3, nad6_codon3	GTR+I+G+X
	P15	nad1_codon1	GTR+I+G+X
	P16	nad1_codon2	GTR+I+G+X
	P17	nad1_codon3, nad5_codon3, nad4_codon3	GTR+I+G+X
	P18	nad2_codon1	GTR+I+G+X
	P19	nad3_codon2, nad2_codon2	GTR+I+G+X
	P20	nad2_codon3	GTR+I+G+X
	P21	nad3_codon1	GTR+I+G+X
	P22	nad4L_codon1	GTR+I+G+X
	P23	nad4L_codon2	GTR+G+X
	P24	nad4L_codon3	HKY+G+X
	P25	nad4_codon1, nad5_codon1	GTR+I+G+X
	P26	nad4_codon2	GTR+I+G+X
	P27	nad5_codon2	GTR+I+G+X
	P28	nad6_codon2	GTR+I+G+X
	P29	rrnL	GTR+I+G+X
	P30	rrnS	GTR+I+G+X
PCGs123+rRNAs-ML	P1	atp6_codon1, cytb_codon1	GTR+F+I+G4
	P2	cox2_codon2, atp6_codon2	TVM+F+I+G4
	P3	atp6_codon3, atp8_codon3	TN+F+G4
	P4	nad6_codon1, atp8_codon1	HKY+F+I+G4
	P5	atp8_codon2	TIM2e+G4
	P6	cox1_codon1	GTR+F+I+G4
	P7	cox1_codon2	GTR+F+I+G4
	P8	cox1_codon3	TIM3+F+G4
	P9	cox2_codon1	GTR+F+I+G4

P10	cox2_codon3, cox3_codon3	TVM+F+I+G4
P11	cox3_codon1	GTR+F+I+G4
P12	cox3_codon2	GTR+F+I+G4
P13	cytb_codon2	GTR+F+I+G4
P14	nad3_codon3, cytb_codon3, nad6_codon3	HKY+F+ASC+G4
P15	nad1_codon1	TVM+F+I+G4
P16	nad1_codon2	GTR+F+I+G4
P17	nad1_codon3, nad5_codon3, nad4_codon3	TPM2+F+G4
P18	nad2_codon1	TIM2+F+I+G4
P19	nad3_codon2, nad2_codon2	TVM+F+I+G4
P20	nad2_codon3	HKY+F+G4
P21	nad3_codon1	GTR+F+I+G4
P22	nad4L_codon1	TVM+F+G4
P23	nad4L_codon2	TVM+F+G4
P24	nad4L_codon3	TPM3u+F+ASC+G4
P25	nad4_codon1, nad5_codon1	GTR+F+I+G4
P26	nad4_codon2	TIM2+F+I+G4
P27	nad5_codon2	GTR+F+I+G4
P28	nad6_codon2	TVM+F+I+G4
P29	rrnL	GTR+F+G4
P30	rrnS	GTR+F+I+G4

Table S3. Mitochondrial genome structure of *Cuspidevia jaechi*.

Gene	Direction	Location	Size	Anticodon	Codon		Intergenic Nucleotides*
					Start	Stop	
trnI	F	1-66	66	32-34 GAT			
trnQ	R	64-132	69	100-102 TTG			-3
trnM	F	133-201	69	164-166 CAT			0
ND2	F	202-1230	1029		ATA	TAA	0
trnW	F	1283-1348	66	1306-1308 TCA			52
trnC	R	1341-1403	63	1372-1374 GCA			-8
trnY	R	1403-1467	65	1435-1437 GTA			-1
COX1	F	1460-3004	1545		ATT	TAA	-8
trnL2	F	3000-3063	64	3029-3031 TAA			-5
COX2	F	3065-3749	685		ATG	T-	1
trnK	F	3750-3820	71	3780-3782 CTT			0
trnD	F	3820-3885	66	3851-3853 GTC			-1
ATP8	F	3886-4041	156		ATC	TAA	0
ATP6	F	4035-4709	675		ATG	TAA	-7
COX3	F	4709-5495	787		ATG	T-	-1
trnG	F	5496-5560	65	5526-5528 TCC			0
ND3	F	5561-5914	354		ATT	TAG	0
trnA	F	5913-5977	65	5942-5944 TGC			-2
trnR	F	5977-6042	66	6006-6008 TCG			-1
trnN	F	6043-6107	65	6074-6076 GTT			0
trnS1	F	6108-6174	67	6133-6135 TCT			0
trnE	F	6175-6239	65	6205-6207 TTC			0
trnF	R	6238-6302	65	6269-6271 GAA			-2

ND5	R	6303-8025	1723		ATT	T-	0
trnH	R	8026-8090	65	8058-8060 GTG			0
ND4	R	8091-9428	1338		ATG	TAA	0
ND4L	R	9422-9703	282		ATA	TAA	-7
trnT	F	9712-9775	64	9743-9745 TGT			8
ND6	F	9785-10300	516		ATT	TAA	9
trnP	R	10301-10365	65	10334-10336 TGG			0
CytB	F	10369-11511	1143		ATG	TAG	3
trnS2	F	11510-11576	67	11541-11543 TGA			-2
ND1	R	11595-12545	951		TTG	TAG	18
trnL1	R	12547-12609	63	12578-12580 TAG			1
l-rRNA	R	12610-13907	1298				0
trnV	R	13908-13978	71	13944-13946 TAC			0
s-rRNA	R	13979-14756	778				0
control region		14757-16309	1553				0

Table S4. Mitochondrial genome structure of *Grouvellinus longiusculus.*

Gene	Direction	Location	Size	Anticodon	Codon		Intergenic Nucleotides*
					Start	Stop	
**trnI**	**F**	**1-66**	**66**	**31-33 GAT**			
trnQ	R	64-132	69	100-102 TTG			-3
trnM	F	140-209	70	171-173 CAT			7
ND2	F	210-1238	1029		ATA	TAA	0
trnW	F	1259-1325	67	1289-1291 TCA			20
trnC	R	1318-1378	61	1348-1350 GCA			-8
trnY	R	1378-1441	64	1409-1411 GTA			-1
COX1	F	1434-2978	1545		ATT	TAA	-8
trnL2	F	2974-3039	66	3003-3005 TAA			-5
COX2	F	3041-3725	685		ATG	T-	1
trnK	F	3726-3796	71	3756-3758 CTT			0
trnD	F	3796-3861	66	3826-3828 GTC			-1
ATP8	F	3862-4017	156		ATT	TAA	0
ATP6	F	4011-4685	675		ATG	TAA	-7
COX3	F	4685-5471	787		ATG	T-	-1
trnG	F	5472-5536	65	5502-5504 TCC			0
ND3	F	5534-5890	357		ATA	TAG	-3
trnA	F	5889-5954	66	5918-5920 TGC			-2
trnR	F	5954-6020	67	5983-5985 TCG			-1
trnN	F	6021-6085	65	6052-6054 GTT			0
trnS1	F	6086-6152	67	6111-6113 TCT			0
trnE	F	6153-6217	65	6183-6185 TTC			0
trnF	R	6216-6281	66	6247-6249 GAA			-2
ND5	R	6282-8004	1723		ATT	T-	0
trnH	R	8005-8071	67	8037-8039 GTG			0
ND4	R	8073-9410	1338		ATG	TAA	1
ND4L	R	9404-9691	288		ATG	TAA	-7
trnT	F	9694-9757	64	9724-9726 TGT			2
ND6	F	9782-10282	501		ATT	TAA	24

trnP	R	10283-10347	65	10316-10318 TGG			0
CytB	F	10351-11493	1143		ATG	TAG	3
trnS2	F	11492-11557	66	11524-11526 TGA			-2
ND1	R	11580-12530	951		TTG	TAG	22
trnL1	R	12532-12596	65	12565-12567 TAG			1
l-rRNA	R	12597-13902	1306				0
trnV	R	13903-13972	70	13939-13941 TAC			0
s-rRNA	R	13973-14748	776				0
control region		14749-16291	1543				0

Table S5. Mitochondrial genome structure of *Stenelmis punctulata.*

Gene	Direction	Location	Size	Anticodon	Codon		Intergenic Nucleo-tides*
					Start	Stop	
trnI	F	1-67	67	31-33 GAT			
trnQ	R	63-138	76	100-102 TTG			-5
trnM	F	138-206	69	168-170 CAT			-1
ND2	F	207-1235	1029		ATA	TAA	0
trnW	F	1254-1319	66	1284-1286 TCA			18
trnC	R	1312-1372	61	1342-1344 GCA			-8
trnY	R	1371-1436	66	1403-1405 GTA			-2
COX1	F	1428-2972	1545		ATT	TAA	-9
trnL2	F	2967-3033	67	2997-2999 TAA			-6
COX2	F	3034-3718	685		ATG	T-	0
trnK	F	3718-3790	73	3759-3761 CTT			-1
trnD	F	3789-3855	67	3821-3823 GTC			-2
ATP8	F	3856-4011	156		ATC	TAA	0
ATP6	F	4005-4679	675		ATG	TAA	-7
COX3	F	4679-5465	787		ATG	T-	-1
trnG	F	5465-5530	66	5496-5498 TCC			-1
ND3	F	5527-5883	357		ATA	TAG	-4
trnA	F	5882-5946	65	5911-5913 TGC			-2
trnR	F	5946-6011	66	5975-5977 TCG			-1
trnN	F	6011-6077	67	6042-6044 GTT			-1
trnS1	F	6078-6144	67	6103-6105 TCT			0
trnE	F	6145-6210	66	6174-6176 TTC			0
trnF	R	6207-6275	69	6239-6241 GAA			-4
ND5	R	6275-8000	1726		ATA	T-	-1
trnH	R	7998-8061	64	8029-8031 GTG			-3
ND4	R	8062-9397	1336		ATG	T-	0
ND4L	R	9391-9678	288		ATG	TAA	-7
trnT	F	9681-9744	64	9711-9713 TGT			2
ND6	F	9765-10274	510		ATA	TAA	20
trnP	R	10275-10339	65	10307-10309 TGG			0
CytB	F	10343-11485	1143		ATG	TAG	3

trnS2	F	11484-11551	68	11515-11517 TGA			-2
ND1	R	11569-12519	951		TTG	TAG	17
trnL1	R	12521-12584	64	12549-12551 TAG			1
l-rRNA	R	12585-13880	1296				0
trnV	R	13881-13950	70	13917-13919 TAC			0
s-rRNA	R	13951-14727	777				0
control region		14728-15480	753				0

Table S6. Substitution Saturation test of PCGs12+rRNAs matrix.

Nu-mOTU	Iss	Iss.cSym	T	DF	P	Iss.cAsym	T	DF	P
4	0.286	0.856	107.539	9124	0.0000	0.846	105.620	9124	0.0000
8	0.292	0.845	94.757	9124	0.0000	0.764	80.842	9124	0.0000
16	0.290	0.846	92.555	9124	0.0000	0.678	64.536	9124	0.0000
32	0.301	0.816	83.388	9124	0.0000	0.571	43.746	9124	0.0000

Table S7. Substitution Saturation test of PCGs123+rRNAs matrix.

NumOTU	Iss	Iss.cSym	T	DF	P	Iss.cAsym	T	DF	P
4	0.392	0.859	96.990	12764	0.0000	0.847	94.466	12764	0.0000
8	0.398	0.844	85.777	12764	0.0000	0.761	69.722	12764	0.0000
16	0.399	0.852	84.450	12764	0.0000	0.675	51.420	12764	0.0000
32	0.403	0.819	76.595	12764	0.0000	0.572	31.199	12764	0.0000

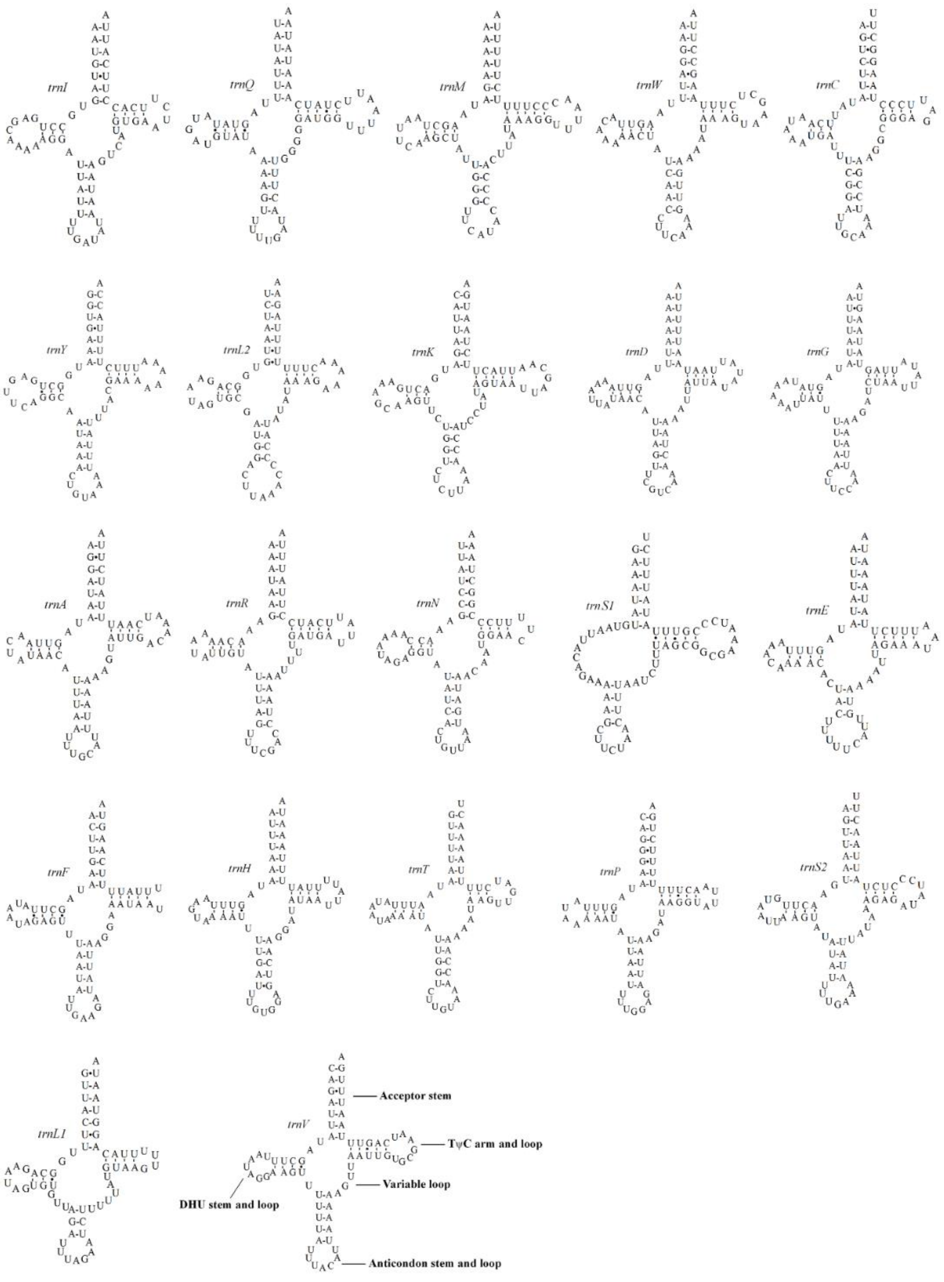

Figure S1. Predicted secondary structure of tRNAs in *Cuspidevia jaechi*.

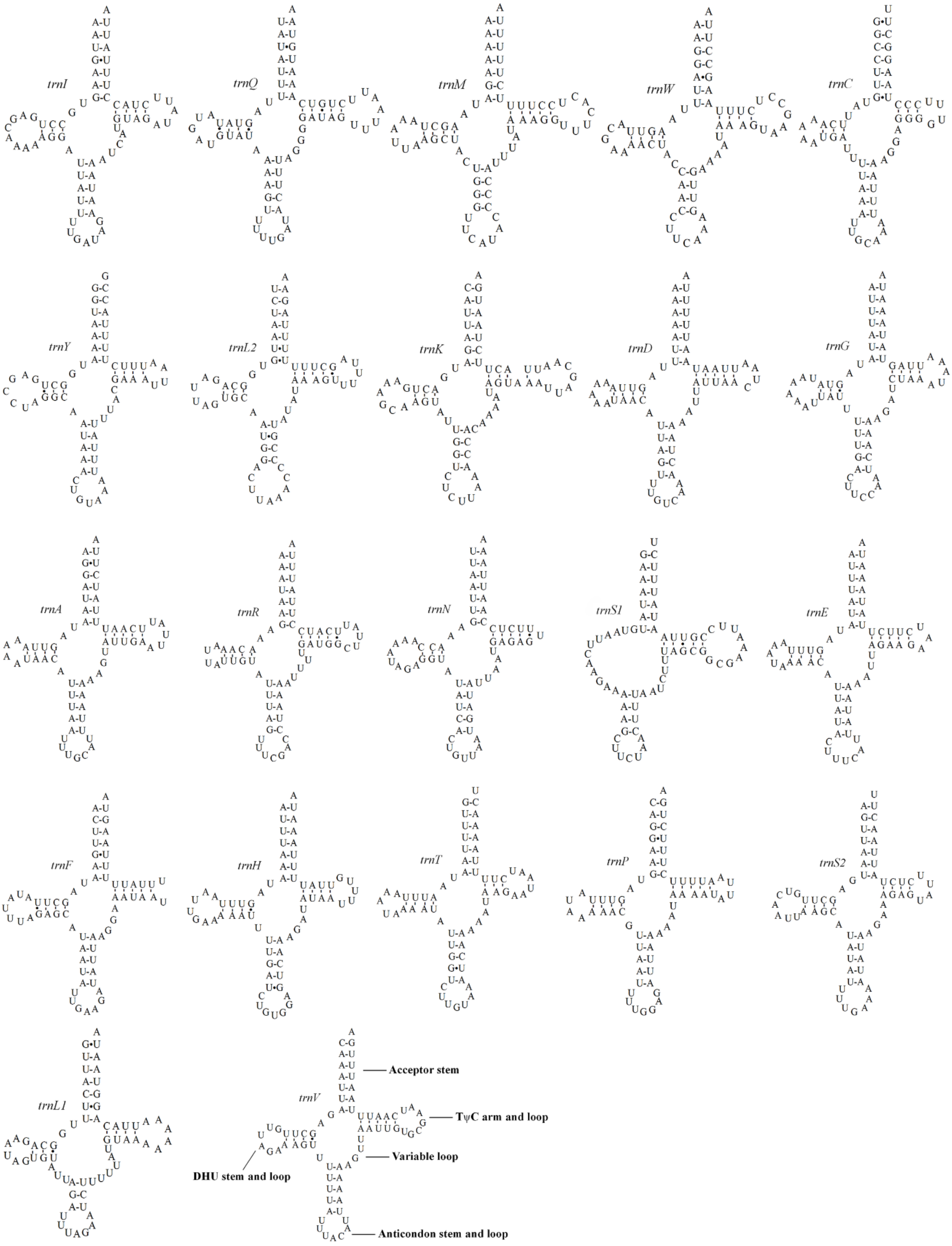

Figure S2. Predicted secondary structure of tRNAs in *Grouvellinus longiusculus*.

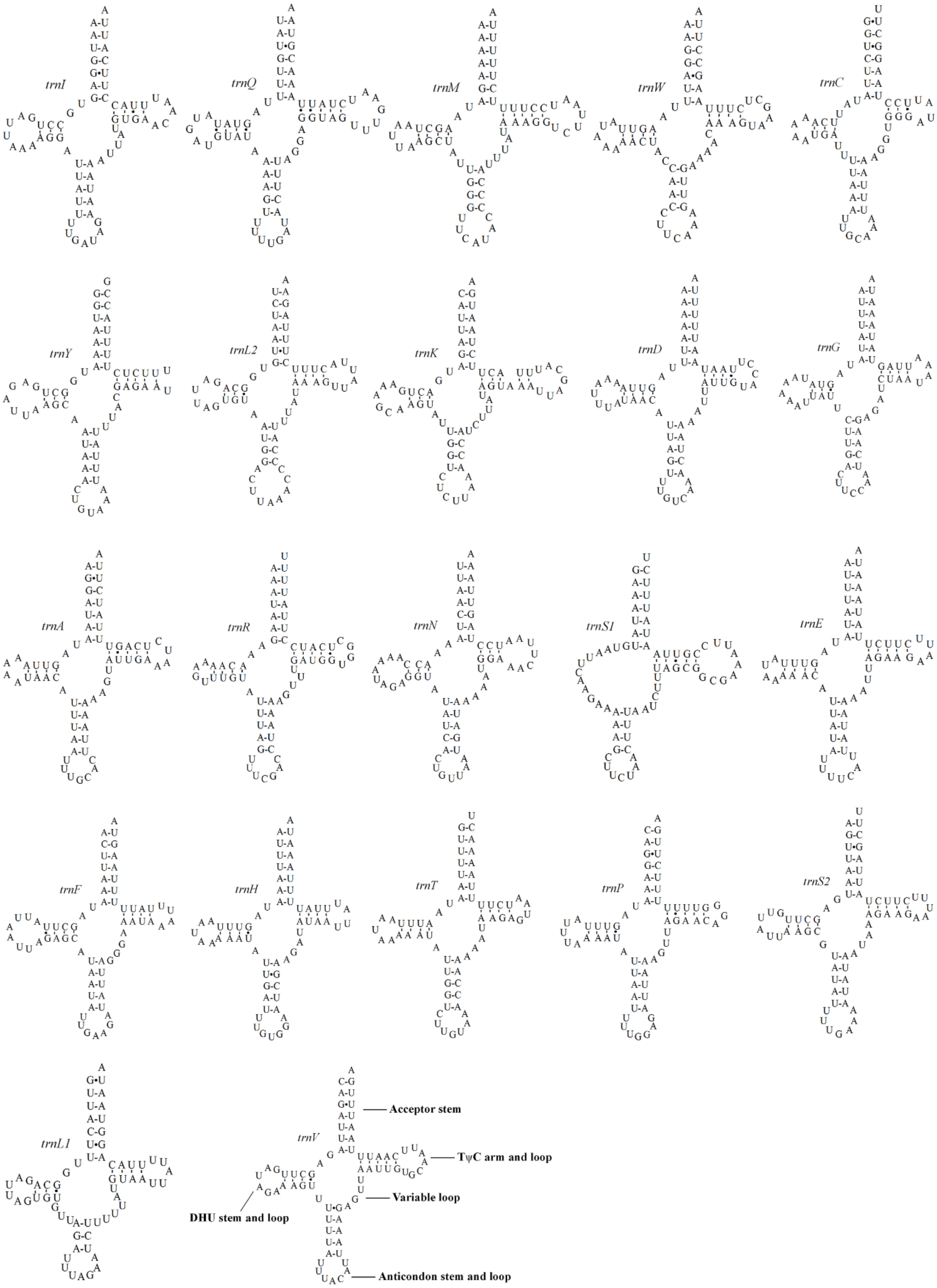

Figure S3. Predicted secondary structure of tRNAs in *Stenelmis punctulata.*

s-rRNA – 14,756 bp

ACCCATATCAATCAAAAAAACATGTTAATTCTATTAATTAATAAAATTAAAGTAAACAATCCTGACTTAGTAAA

46 bp repeat element

CTATCCCCAGTTAAGCCCTTCTACAAGTAAGTAACTTTTTACTCAATAGAAGACAAAACCAATGGATTTTTACT

CAATAAGTAGAGATATCCAGTGCAACTCAGTTAACTTTAATTGAAAGTTAGTAATTTCAAGTCAAGAACTAAGA

CAAAATTTCTTGACCATAAAAAATTAATTAATATTATCCCTAAATTAATTAACCCAGACTTAAATTCTAGCCTTTTA

Poly-A element

AAGCTAAATCTAAAAATTAAATAAACTAAAATGAAAACAACCGTTAATTACCCCTAATTAACATAATGATTTCAA

ATTAATCAAGTAAATTAATAAATCTCAAGCAAATTCAATAACAAAATCAAACTAAGCCCCATAACTCATTCAACT

CCCATCTCATACTCAAAATTCCCGCATCACCATTAATATTTAATTGACTTTAAATTTAAAATTATATTTATATATAAA

17 bp repeat element

ATTACTTAACAAATAAATAATATCTTATTTTTATATATAAACAATAATTATAAAATAAATCCAGTAAGTTTATTTTTT

Poly-T element

TAAAATAAAACTTTAAACTCAAATTAATCTTAAAAAACATGTTAATTCTATTAATTAATAAAATTAAAGTAAACAA

46 bp repeat element

TAAAAAAAAACTAAAATATTAATTAAACAATTATAAAAAAGCTATTTATATTAAAATAAGTTTCCATAATTGAGTC

Poly-A element

TATTTTTTAATAGACTTAAATAAGGTAACAATAAAATTGATAGAACATAATTTAAACCCTTTTTTTTTTAGAAATC

Poly-T element

AGTAAAAATATTGACTTAGTAAATAATCTCACTAAATAAATAGTTTATGATTATATATTGCATTTAACATTCATTTAA

TTAGTTAATAATAACTTACTGGTCTAAACTGTTATCTATCTATTTAAAATTAATTTTATTATATTTTTCAATTAAACA

ATAATCAATAATTCCTCCAATATTGATTATTATTTAATTGAAAGATATAATTATATAAGCATATATATATATATAAATAT

Macrosatellite (AT)$_n$ element

AATAATAGGTATAAGGTTCACTACCGTAAACTAAAATTTTACATATTAATTCTCTCTCTCTCTCAAATATTAAACTT

Macrosatellite (CT)$_n$ element

GTAGATATTAAGAAGGCAATCATAGACGAATTGAACACGTTTTAACTGATAGATAGACTTTTTTTTTTAACATTTC

Poly-T element

ATTTAAAATTATATTTAAATTAAATCATTATACATATATATATAATGTATGTATGCATTAATATATATATATATATATCTA

17 bp repeat element Macrosatellite (AT)$_n$ element Macrosatellite (AT)$_n$ element

TAATAATTTAATATATATATATGCATATATATTATGTATAATTTAAACATTTATTAACAAGAAAAAAATTAAAACTA

Macrosatellite (AT)$_n$ element Poly-A element

CACATTTATAATTTAGACCAAATTTACAATTTCAATTAGTCTCAAATTATCAACCAAAACTCATAAATGAAGAAAT

AATACCTTATCAACCAATAATTTATTAATAAAGGAATATAAATAAATAACAAAATTCCACATGTCAAAAAATTTAA

TGTCCCATTCAAAGTTTT 16,309 bp – trnI

A

s-rRNA – 14,748 bp

TCTAAATAAATAAAATTATGACATAATAAATATAAATTAACAAAATCTCAAGGAAGAAATTTATCCTTTAGTAAGG

TAGGTCCAGTCAGGCCCTCTTAAAGATATTTATCTCTAAGAGACCTAACCAGTGGATTTTAAGTACAAAACAAG

AGATCCAGGAAACTTAATTAACTTTAAATGAAAGTTAGTAATTTAAAAGCCAAAAGCCAGAATATGCTTTTAAC

CCTAATTGACTTATTAATAAGTCAAGTGAACGACATTAGATTGAAAACAATTAAACCCAAAATCAAAATTATATA

AACTGAACTATTAAAATTAAATAAAAATTTTTGCCCCTTAATGCATAAGCATCAAAAAAATTTCTAATCTAATATT

14 bp repeat element Poly-A element

AATAATCAAAAAAAAATAAGCCACAAAAACTAAATAATTTATTTTTACCCCAAAAATAAATTAAAATTAATGTAAC

Poly-A element

TCAAAATTTTTGAGCCCCAATCCCGTTTATATAAAATTTAAATAATTGAATCTAAGGTTATTGATATTAAAAGTAAT

TTGTCTAAAAAACAATATTCTGTACTATTATAAAACCAATTAACCACTAACTTGTTCGAAAAAAATAAAACTTTG

Poly-A element

TATAAACAATAAATATAATACGACATAATTAATTTATAATAACTAATTTTATAATAATTAAATAATTATTAATAAAAAT

CATTAGTGATTAAATATAATATAAAATTAAAATTAAATATAATTAAAAAAAAAAATTAATAAATTAAGAAATTTCAA

14 bp repeat element Poly-A element

AAAGAATTAATAAAGAATAACTAATTAAAAACTACAGTTTTTTTTTTTTTTATATATAAAAATTAATTTTTTGGTAA

Poly-T element 13 bp repeat element

ATATTGAAATACAATAAATAATTTATGATTATATTATATATACCTTTTATTTAATATATAGTTAAAATTATATTTAAATA

TTAGTATTTTATATTATAATAAATAAATAATCTAATTATATTTTAAATTGAATAATTATATATTAATCACACTATATTGA

CTATTATTTAATTTAAAATTAATTATATATATATATTTATATATTAATAAATGTAATATATTATATAAGATTCACTACCGA

Macrosatellite (AT)$_n$ element

TAATTAAAATATGAATATCCCATACTCTCTCTCTTTTTAATATTAAACCTGTAGATATCCATAAAATAATCATATATT

Macrosatellite (CT)$_n$ element

CATTGAACACGGTTTATTTAATAGATATCCTTTTTTTGTTCATATTATAGTCAAGTATCTTTATTTATATAATCAATAT

Poly-T element

ATATTTATTTAAATTATATGTTGCATTATAATATATATATATATATAAATTTTAATTTAATGTATAATACATGTATATGTT

Macrosatellite (AT)$_n$ element

AGTATATTCATTAAATATTTATATAAAGAGAAAAGTTCTAAAAGTGCTACCTAACACTTTTAGAACAATTGGAGAT

TTTGGGGTATGCACAAATTAAATCTTTTTTTTTTCGATTTTTTTTTTTAAGATTAATGTCACGGCTATATTTAACCT

Poly-T element Poly-T element

AAATTTTATAAACAAACAAATTAATAAGAAATAAAAGTTATATATGTCCAAAAAATTAATTTTCAATATTAAGTTT

13 bp repeat element

T 16,291 bp – trnI

B

s-rRNA – 14,727 bp

GATAAATAAATAAATAAATAACAAACAAAATAAAATATTATTAAAACGCTAAATATCAAAAAATAATAATTAATTT

8 bp repeat element 13 bp repeat element

AAATTAACGACTACATAAAGAAAACTTTAATTAAATAAAATATTAAATTATAATTACTAAAATGTTTTATTAATATC

13 bp repeat element 12 bp repeat element

AATTACTTTTTTTTTTGATGATTAATATATAATTTAAAAAAAGATTTTCTTATATAAATAATTTATTAATCTATAAGCT

Poly-T element Poly-A element

AAATATATTATATATATACACATAATTAATACTTTCAATATGTTATATATTAGACAATAATTTTATATATTTATATATTAA

Macrosatellite (TA)$_n$ element

TATTTACTCATAAATAAGGTTCACTATATAAAATTAAGAGTTTATATCCCCATATTTTTCTCTCTTTCATATATTAAA

CTCTTAGATATTTATACGATAATCATAGATAAGATAAACAAAGTTTAACTAATAGATAGACCCTTTTATAACAATAA

ATTGAATAATATATAAAAATTAAATTATTATATATAATTAAATATTATTCAATTTTGGAAATAATTATATAACAGGACT

TACTTTATAATAATATTTAAATTTACATGTATATTATTTTATGTAAACATTTATTAGAAAAAAAAAGAAAATCCCCA

Poly-A element

AACCAAACTATCACGAAGATATATTAAATTCATATTTTTTGAAATTAATTTTAATAAATTAACTAAATAAATAAAAT

ATATAAAACAACAAAATAATGTTTTATTATTTTTTGTTTAAATAAAAGTTTT 15,480 bp – trnI

12 bp repeat element

C

Figure S4. Control region of *C. jaechi*, *G. longiusculus* and *S. punctulata*. (**A**) *C. jaechi*, (**B**) *G. longiusculus*, (**C**) *S. punctulata*.
